# The contribution of the vascular architecture and cerebrovascular reactivity to the BOLD signal formation across cortical depth

**DOI:** 10.1162/imag_a_00203

**Published:** 2024-06-28

**Authors:** Emiel C.A. Roefs, Wouter Schellekens, Mario G. Báez-Yáñez, Alex A. Bhogal, Iris I.A. Groen, Matthias J.P. van Osch, Jeroen C.W. Siero, Natalia Petridou

**Affiliations:** Department of Radiology, Center for Image Sciences, University Medical Center Utrecht, Utrecht, Netherlands; C.J. Gorter MRI Center, Department of Radiology, Leiden University Medical Center, Leiden, Netherlands; Donders Centre for Cognitive Neuroimaging, Radboud UMC, Nijmegen, Netherlands; Departement of Psychology, New York University, New York, NY, USA; Video & Image Sense Lab, Institute for Informatics, University of Amsterdam, Amsterdam, Netherlands; Spinoza Centre for Neuroimaging, Amsterdam, Netherlands

**Keywords:** gradient-echo BOLD, hypercapnia, hyperoxia, laminar fMRI, spin-echo BOLD, normalization

## Abstract

Assessment of neuronal activity using blood oxygenation level-dependent (BOLD) is confounded by how the cerebrovascular architecture modulates hemodynamic responses. To understand brain function at the laminar level, it is crucial to distinguish neuronal signal contributions from those determined by the cortical vascular organization. Therefore, our aim was to investigate the purely vascular contribution in the BOLD signal by using vasoactive stimuli and compare that with neuronal-induced BOLD responses from a visual task. To do so, we estimated the hemodynamic response function (HRF) across cortical depth following brief visual stimulations under different conditions using ultrahigh-field (7 Tesla) functional (f)MRI. We acquired gradient-echo (GE)-echo-planar-imaging (EPI) BOLD, containing contributions from all vessel sizes, and spin-echo (SE)-EPI BOLD for which signal changes predominately originate from microvessels, to distinguish signal weighting from different vascular compartments. Non-neuronal hemodynamic changes were induced by hypercapnia and hyperoxia to estimate cerebrovascular reactivity and venous cerebral blood volume ($CBVv_{O_{2}}$). Results show that increases in GE HRF amplitude from deeper to superficial layers coincided with increased macrovascular$CBVv_{O_{2}}$.$CBVv_{O_{2}}$-normalized GE-HRF amplitudes yielded similar cortical depth profiles as SE, thereby possibly improving specificity to neuronal activation. For GE BOLD, faster onset time and shorter time-to-peak were observed toward the deeper layers. Hypercapnia reduced the amplitude of visual stimulus-induced signal responses as denoted by lower GE-HRF amplitudes and longer time-to-peak. In contrast, the SE-HRF amplitude was unaffected by hypercapnia, suggesting that these responses reflect predominantly neurovascular processes that are less contaminated by macrovascular signal contributions.

## 1 Introduction

Since the introduction of blood oxygenation level-dependent (BOLD) functional magnetic resonance imaging (fMRI) byOgawa et al. (1990;Kwong et al., 1992), it has become one of the most widely used tools to investigate brain function noninvasively. Recent advancements in sequence design and MRI hardware technologies in combination with high-field (≥7T) MRI have facilitated high resolution and dynamic imaging of hemodynamic signals associated with neuronal activity (Dumoulin et al., 2018;Petridou & Siero, 2019;Petridou et al., 2013). These developments have offered the potential to perform fMRI measurements at submillimeter spatial and/or subsecond temporal resolution, which have facilitated investigations at the laminar or columnar level in the cerebral cortex. However, the BOLD signal reflects neuronal activity indirectly, through local changes in the concentration of deoxyhemoglobin ([dHb]) in the blood by alterations in metabolic rate of oxygen consumption (CMRO_2_), local cerebral blood flow (CBF), and volume (CBV). These hemodynamic responses support the increase in cerebral metabolic rate of oxygen consumption (CMRO_2_) following neuronal activation. In addition to the hemodynamic basis of the BOLD signal, the cortical vascular architecture also plays a role in the BOLD signal formation.

For instance, in the human cortex, the volume of venous macrovessels increases toward the pial surface, suggesting more venous weighting toward the pial surface (Duvernoy et al., 1981). In comparison with the intracortical vessels, pial vessels are more sparse and larger in diameter (>100 µm) (Duvernoy et al., 1981). Further, the microvascular density can vary across cortical depth and across brain regions but is much more uniform than the macrovasculature (Duvernoy et al., 1981;Weber et al., 2008). Duvernoy et al. showed a slight increase in capillary density in layer IV in the medial frontal gyrus and the Brodmann area 17 (Bell & Ball, 1985;Duvernoy et al., 1981,^1983^;Weber et al., 2008). Besides the variable vessel density and size, which implies different resting CBV (and CBF), heterogeneous red blood cell fraction, thereby differences in dHb concentration and associated susceptibility effects (Haacke et al., 2004;Ogawa et al., 1990), also impacts the BOLD amplitude and timing across cortical depth (Schellekens et al., 2023;Siero et al., 2011,^2013^). Deoxygenated blood flows from capillaries into venules, ascending veins, and eventually to pial veins (Hirsch et al., 2012;Schmid et al., 2019). This translates to larger amplitude, width, and delay of the BOLD response toward the pial surface. These characteristics mainly reflect differences in vascular architecture rather than differences in neuronal activation patterns (Petridou & Siero, 2019;Petridou et al., 2013). Notably, arterioles dilate within hundreds of milliseconds from stimulus onset, followed by upstream dilation of superficial arteries (Tian et al., 2010), whereas venous vessels dilate passively, much slower, and only with prolonged stimulation (Drew, 2019;Huo et al., 2015;Yu et al., 2016). These spatial differences in macro- and microvascular dilation characteristics will directly translate to a delayed hemodynamic response in the GM toward the pial surface as has been shown for the BOLD signal in the white matter (WM) already (Bhogal, 2021). Understanding how the different compartments of the vascular architecture contribute to the temporal and spatial features of the BOLD signal is, therefore, critical for accurate interpretation of fMRI data. Particularly for laminar-fMRI studies that aim to resolve neuronal-induced signals at different cortical depths (Ahveninen et al., 2016;Fracasso et al., 2018;Guidi et al., 2016;Polimeni et al., 2010;Siero et al., 2013), this is important because of the variable vascular architecture across cortical depth (Duvernoy et al., 1983;Hirsch et al., 2012;Schmid et al., 2019).

The aim of our study was to investigate how vascular properties may influence signals that are normally ascribed to neurovascular coupling. To achieve this, we acquired high-resolution, subsecond BOLD measurements at 7T that facilitated the interrogation of laminar-specific responses. We estimated the contributions of pial veins, intracortical veins, and microvessels to the BOLD response upon a brief visual stimulus while simultaneous vasoactive stimuli informed on more pure vascular properties. Throughout this article, we use the term microvasculature for the combined network of arterioles, capillaries, and venules. Different vascular compartments were targeted by employing different scan sequences: spin echo (SE) echo-planar-imaging (EPI) BOLD is weighted predominantly to the microvasculature (Budde et al., 2014;Duong et al., 2003;Uludağ et al., 2009;Yacoub et al., 2003), and is, therefore, used as tool to study the microvasculature, while gradient echo (GE)-BOLD is sensitive to all vessel sizes (Budde et al., 2014;Duong et al., 2003;Menon, 2012;Uludağ et al., 2009). To separate neuronal or metabolic-induced changes in the BOLD signal from vasoactive or chemical-induced changes (Ainslie & Duffin, 2009), neurovascular HRFs, the change in BOLD signal induced by neuronal activation, were estimated following very brief visual stimulations of 200 ms. Concurrently with the visual stimuli, non-neuronal hemodynamic changes were induced by targeted end-tidal CO_2_(hypercapnia) and O_2_(hyperoxia) manipulations in arterial blood gases to estimate cerebrovascular reactivity (CVR) and relative venous baseline blood volume ($CBVv_{O_{2}}$). Hypercapnia results in dilation of vessels leading to increased CBF and CBV and thus a decrease in [dHb]. However, hyperoxia does not notably change CBF nor CBV for short stimuli as used here (Chiarelli et al., 2007), but has a direct positive effect on venous oxygen saturation (SO_2_).

In this paper, vasoactive stimuli will inform on purely vascular effects in the BOLD response, while short event-related visual stimuli will be used to estimate the neurovascular component during these gas challenges. A hyperoxia task is used to estimate$CBVv_{O_{2}}$as a proxy for baseline venous blood volume for normalization of the HRF. SE- and GE-BOLD were compared to differentiate between microvasculature and all venous vessel types.

## 2 Materials and Methods

### 2.1 Subjects

Eleven healthy volunteers (N = 11, age range 18-42 years, mean = 24.3 years, female = 8) participated in this study after giving written informed consent. All subjects declared to have no form of visual impairment nor suffering from pulmonary conditions. The experimental protocol was approved by the ethics committee of the University Medical Center Utrecht (UMCU) and conducted according to the principles of the 2013 Declaration of Helsinki and the Dutch Medical Research Involving Human Subjects Act.

### 2.2 Scan protocol

Scanning was performed on a 7T Philips Achieva scanner (Philips Healthcare, Best, the Netherlands) with two 16-channel high-density surface receive arrays (MRCoils BV, the Netherlands,Petridou et al. (2013)). BOLD fMRI scans were acquired employing GE-EPI and SE-EPI. The session was divided into two runs of GE-BOLD and two SE-BOLD runs. GE-EPI fMRI data were collected with SENSE-factor = 4.0, EPI-factor = 31, repetition time/echo time (TR/TE) = 850/27 ms, flip-angle (FA) = 50 degrees, number of slices = 7, field of view (FOV) = 7 × 128 × 128 mm^3^(anterior-posterior, inferior-superior, right-left), covering early visual areas, voxel size = 1.0 mm isotropic. To improve the signal-to-noise ratio (SNR), SE-EPI data were collected at lower spatial resolution with SENSE-factor = 2.0, EPI-factor = 63, TR/TE = 850/50 ms, FA = 90 degrees, number of slices = 5, FOV = 7.5 × 190 × 190 mm^3^, voxel size = 1.5 mm isotropic. Each run consisted of 820 volumes (duration = 697 s). GE-EPI and SE-EPI runs were interleaved. Additionally, EPI images with reversed phase-encoding direction were acquired for both GE and SE. Respiration and heart rate were recorded using a breathing belt and a pulse oximeter attached to the index finger, to calculate the respiration volume per time (RVT) and beats per minute (BPM) (Birn et al., 2008).

T2*-weighted flow-compensated anatomical images with partial brain coverage (80 slices covering the posterior occipital cortex) were acquired using 3D-EPI (Fracasso et al., 2018;Zwanenburg et al., 2011) with spatial resolution of 0.5 mm isotropic, FOV 40 × 160 × 160 mm^3^, TR = 56.0 ms, TE = 30.0 ms. T1-weighted structural images were obtained using MP-RAGE with the same FOV, spatial resolution 0.8 mm isotropic, number of slices = 50, TR/TE = 7.0/2.97 ms. A proton density (PD) weighted scan (TR/TE = 6.1/3.0 ms, FA = 1°) was acquired in order to correct for signal intensity inhomogeneities. Whole brain T1-weighted anatomical data obtained at 3T (spatial resolution = 1.0 mm isotropic, FOV = 232 × 256 × 192 mm^3^, TR/TE = 7.9/4.5 ms) were also available for 5 out of 11 subjects.

### 2.3 Experimental design

During functional data acquisition, subjects performed a visual task while undergoing manipulations in arterial blood gas concentration using a computer-controlled gas delivery system, later referred to as gas challenges. No visual tasks nor gas challenges were performed for the first 30 s to obtain a baseline BOLD signal.

#### 2.3.1 Visual task

**Fig. 1.**
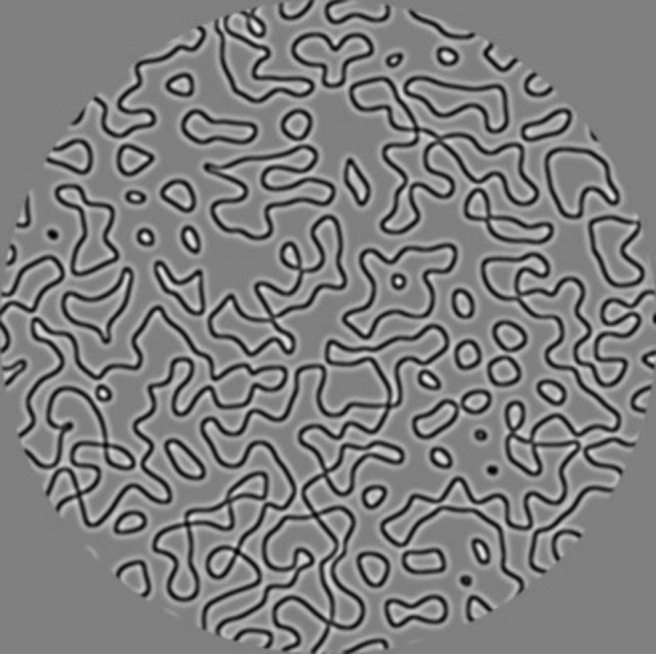
Visual stimulus: Example of visual stimuli presented to the subjects: 71 repetitions (event related), each for a duration of 200 ms interleaved with periods of gray screen.

The visual task consisted of 71 event-related short stimuli with pseudorandom interstimulus interval ranging between 3 and 28 s (right-skewed exponential distribution) during the functional runs with a full duration of 697 s. Each stimulus presentation lasted for 200 ms, interleaved with periods of a gray screen. The visual stimuli were static grayscale randomly oriented contrast images (Fig. 1) windowed with a circular aperture, and were generated in MATLAB (Mathworks, Natick, MA, USA) using PsychToolbox. The randomly oriented contrast was altered for each occurrence. Stimuli were presented via back projection onto a 13.0 × 8.0 cm^2^screen (1920 × 1080 pixels, 60 Hz) at a viewing distance of 37.5 cm and were observed through prism glasses and a mirror. The radius of the stimulus extent was 9.89^°^visual angle. It has been shown using intracranial electrophysiology that no habituation is observed for this stimuli with such short duration (Groen et al., 2022).

#### 2.3.2 Gas challenge

**Fig. 2.**
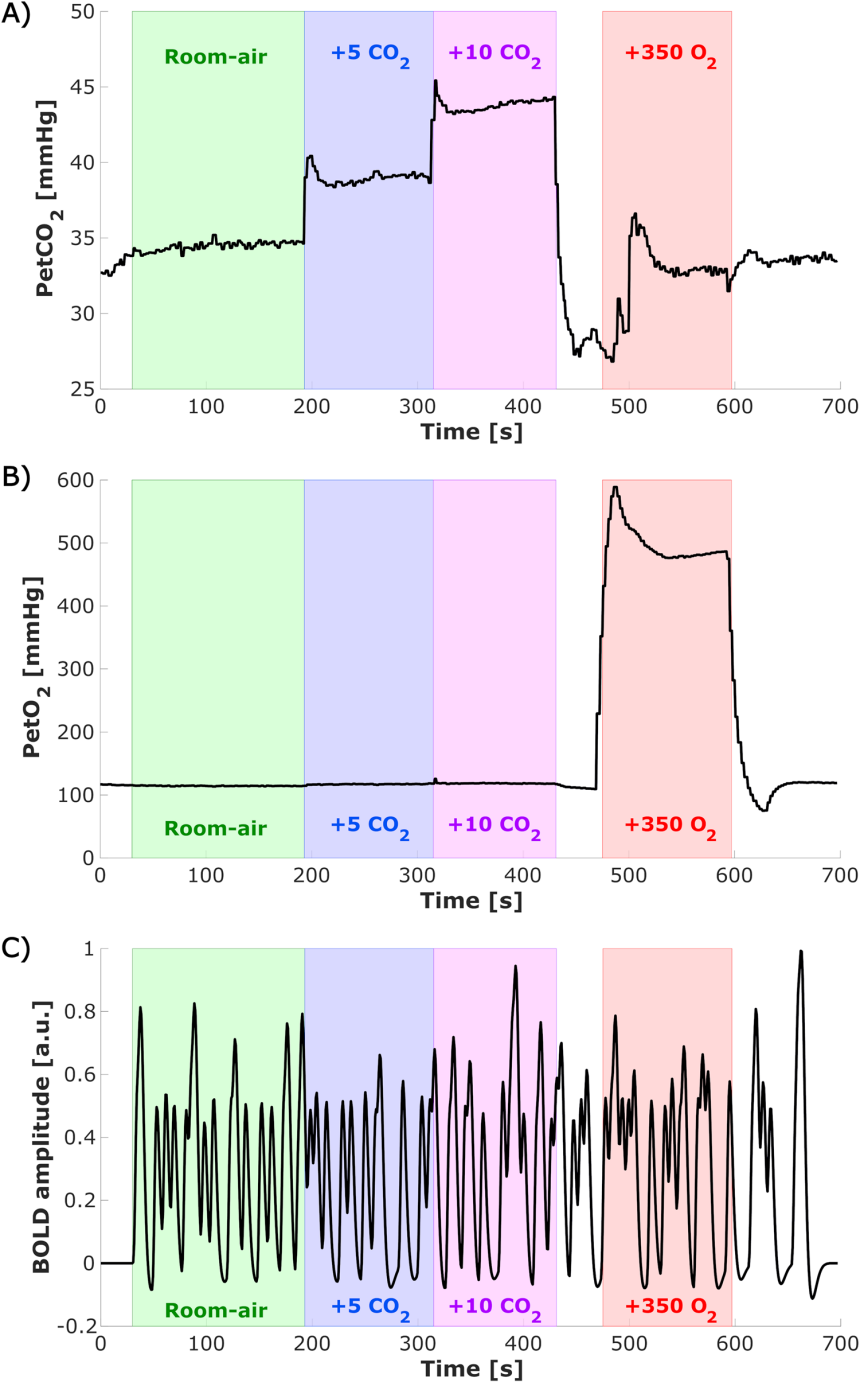
Experimental design: Time series of end-tidal*PetCO*_2_(A) and*PetO*_2_(B) gas conditions measured with the RespirAct for a representative subject, and expected time series of the BOLD responses to the visual task excluding the gas challenges scaled to [-1 1] (C, event-related stimuli convolved with the canonical HRF). Colors indicated the different periods of the gas challenges within this functional run (green = room air, blue = +5 mmHg***PetCO*****_2_**, purple = +10 mmHg***PetCO*****_2_**, red = +350 mmHg*P**etO*****_2_**). These colors are used throughout the rest of the figures to indicate the different gas challenges.

Simultaneously with the visual task, end tidal (Pet)CO_2_and PetO_2_pressure values were targeted using a computer-controlled gas blender and sequential gas delivery system (Third generation RespirAct™, Thornhill Research Inc, Toronto, Canada). Each functional run (total duration of 697 s) consisted of four different parts: (1) a 200 s period with a gas mixture resembling room air, (2) a 120 s period of room-air + 5 mmHg PetCO_2_target, (3) a 120 s period of room-air + 10 mmHg PetCO_2_target, and (4) a 120 s period of room-air + 350 mmHg PetO_2_target (Fig. 2). As a consequence of the longer duration of room-air breathing compared with the gas challenges, more visual stimuli are presented during this period. In total, 5 out of 11 participants also performed similar runs with room-air + 3 and room-air + 8 mmHg PetCO_2_. However, these runs have been excluded for the current analyses, because of the limited number of participants. Two participants were excluded because not all scans were completed, resulting in nine included subjects in this study.

### 2.4 Structural data preprocessing

T1-weighted images were used to segment the cortex in laminae as a measure of cortical depth. T2*-weighted images were used to segment pial veins based on their dark appearance. Throughout this manuscript, the word “laminae” is used rather than “layers” to emphasize that these laminae do not represent architectonic layers distinguishable with histology (e.g., layer IV), but reflect a measure of cortical depth.

#### 2.4.1 Pial vein segmentation

**Fig. 3.**
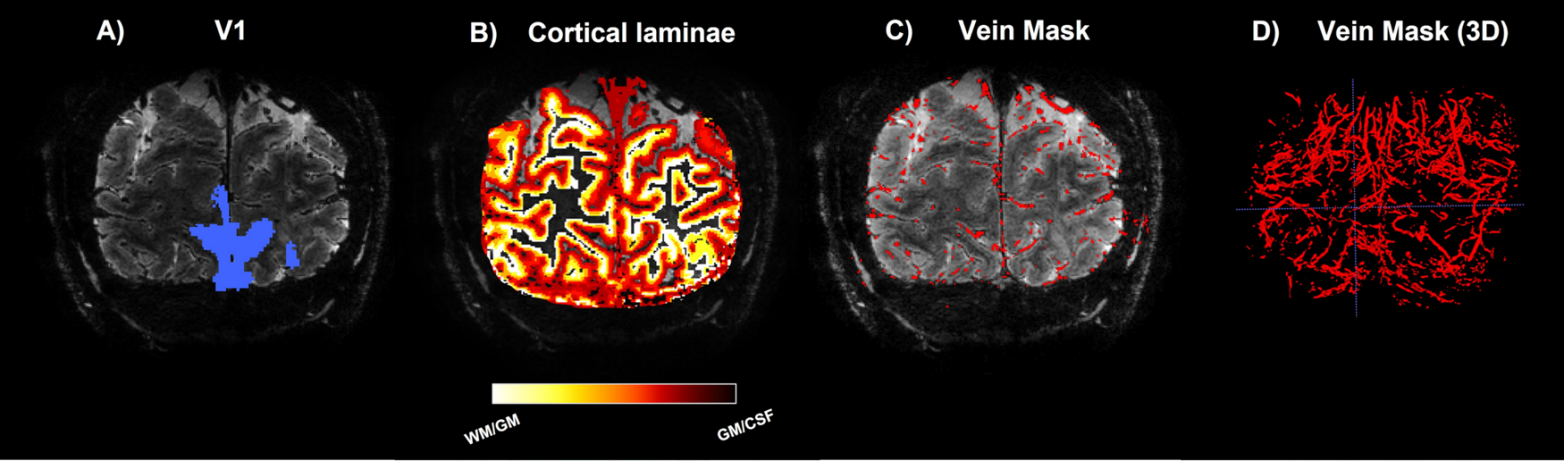
Region of interest definitions: Volumetric maps superimposed on T2*-weighted scan. From left to right: primary visual area definition (A), laminae masks (B), pial vein mask (C), and a 3D render of the pial vein mask (D).

Pial vein segmentation was performed with two tools that were considered complementary since they use a different input (Fig. 3C, D; SupplementaryFig. S1). First, the magnitude image of the T2*-weighted scan was processed with Braincharter (Bernier et al., 2018) with the following settings:*bright vessels = false, alpha = 0.8, beta = 1.0, gamma = 500, and otsu_offset = 1*. Second, a quantitative susceptibility map (QSM) was reconstructed from the phase image of the T2*-weighted scan by Laplacian-based unwrapping, SHARP background filtering (Schweser et al., 2011;Sun & Wilman, 2014), and subsequently employing an iterative rapid two-step dipole inversion method (Kames et al., 2018). The Python package Nighres (Huntenburg et al., 2018) was utilized to segment veins based on susceptibility difference using recursive ridge filtering by using the QSM as input (Bazin et al., 2016;Huck et al., 2019). The union of both tools was taken to produce the final vein map.

#### 2.4.2 Cortical laminae

The T1-weighted scan was corrected for intensity inhomogeneities using the PD-weighted scan (Van de Moortele et al., 2009), and was upsampled to higher resolution (0.3 mm isotropic). The upsampled scan was used to segment the cortical surface using the ANTs (https://github.com/ANTsX/ANTs,Avants et al., 2011) function*antsAtroposN4*. The cortical surface was divided into 20 equivolumetric laminae using the LayNii function LN_GROW_LAYERS (Huber et al., 2021), and subgrouped into 6 laminae masks (Fig. 3B). The segmented veins were subsequently excluded from the laminae masks. Transformation matrices were computed to coregister the previously described vein and laminae masks to the functional images, as all further functional analyses were performed in native functional space.*antsRegistration*was used for a two-pass registration of the anatomical scans with the mean functional time series image.

#### 2.4.3 Probability masks

The laminae mask was generated in higher resolution than the functional data. Because of that, the lower resolution functional voxels often encompass more than 1 anatomically defined cortical depth level as defined inFischl and Dale (2000). To minimize the resulting partial volume effects, a probability-weighted cortical depth estimation was computed followingvan Mourik et al. (2019). This probability mask was created by linearly warping the vein and laminae masks to functional space, giving a probability for each mask to be present in each voxel in functional space. These probabilities were used to create laminae-specific results defined as the weighted average over all voxels. The intersection of the volumetric probability mask (Fig. 3B) and area V1 (Fig. 3A) entails the region of interest.

#### 2.4.4 Visual area localization

The whole-brain structural images acquired at 3T were processed with FreeSurfer (https://surfer.nmr.mgh.harv) to obtain estimates of white matter and pial surfaces (Fischl et al., 2002). These surfaces were then used to generate a surface-based visual area map using the anatomically defined Benson atlas of visual areas (Benson et al., 2014) in Neuropythy (https://github.com/noahbenson/neuropythy) (Benson & Winawer, 2018). From this visual area parcellation, a volumetric map of early visual area V1 was generated using the FreeSurfer tool*mri_surf2vol*(Fig. 3A). Gaps in this volumetric map were filled with an in-house IDL (Exelis Visual Information Solutions, Boulder, Colorado) script. When no 3T scan was available, visual areas were estimated using a nonlinear warp of a standardized Benson atlas and the 7T T1-weighted volume. In this study, only V1 was examined as neuronal responses may differ per visual area, as well as the vascular organization (Fonta & Imbert, 2002;Handwerker et al., 2004;Schmid et al., 2019;Weber et al., 2008;Zheng et al., 1991).

### 2.5 fMRI data preprocessing

All functional scans were corrected for rigid body motion with AFNI*3dvolreg*(Cox, 1996). An EPI acquisition distortion warp was calculated with AFNI*3dQwarp*using the EPI images with opposite phase-encoding directions. This EPI distortion correction and the motion correction were simultaneously applied in a single interpolation step to generate the motion-corrected undistorted functional scans.

### 2.6 fMRI data analysis

First, a general linear model (GLM) was employed to determine the CVR and the$CBVv_{O_{2}}$from, respectively, the hypercapnia and hyperoxia experiments. The$CBVv_{O_{2}}$is obtained from the hyperoxia data similarly as CVR is obtained from the hypercapnia data (%BOLD/mmHg). Since the vessels do not actively react to the hyperoxia stimulus, while the venous oxygenation does increase upon the challenge, we did choose the term$CBVv_{O_{2}}$, since the percentage BOLD change per mmHg O_2_will mainly be dependent on the relative venous CBV and intravenous oxygenation (Blockley et al., 2013). Since the$CBVv_{O_{2}}$as measured with GE and SE will not refer to the same quantity, the sequence is added as a superscript (e.g.,$CBVv_{O_{2}}$) when comparing sequences. A single GLM was used to locate voxels that responded both to the gas challenges and to the visual stimuli (Section 2.6.1). Subsequently, the HRF was estimated for these commonly responding voxels using a finite impulse response (FIR) approach (Section 2.6.2).

#### 2.6.1 Localization of voxels responding to the gas challenge and visual stimuli

Time series were detrended with a discrete cosine set (cutoff frequency = 7*10^-4^Hz) and responding voxels were identified by using a GLM using the end-tidal gas traces (Fig. 2A, B), in ∆mmHg, and visual stimuli timings convolved with a canonical HRF (Fig. 2C) as regressors of interest. The motion parameters from motion correction and physiological parameters (RVT and BPM) were added as nuisance regressors. All nuisance regressors were detrended similarly as the functional time series but were not convolved with a response function. CVR (Poublanc et al., 2015) and the$CBVv_{O_{2}}$were defined as the linear regression coefficient of (%∆BOLD) versus ∆Pet*CO*_2_and ∆Pet*O*_2_, respectively. T-statistics of the hypercapnia and hyperoxia obtained from the GLM were corrected using false discovery rate (FDR) correction (q < 0.05). No FDR correction was performed on the visual task regressor, but a broad selection of visually responsive voxels within the ROI mask was performed for subsequent FIR analysis based on a loose t-statistics threshold of t > 1. The union of the above-described t-statistic maps is used for further analysis.

#### 2.6.2 Voxel-wise HRF estimation

For the commonly responsive voxels to the gas challenges and the visual task, the HRF curve to the visual stimulus during each gas challenge was estimated. First, the time series were high pass filtered with a discrete cosine set (cutoff frequency = 0.01 Hz) and the end-tidal gas traces were additionally regressed out in this step. The HRF curves were estimated with a finite impulse response (FIR) (Bai et al., 2007;Glover, 1999;Goutte et al., 2000), assuming an HRF duration of 20 s for the V1 at 7T (Siero et al., 2013). The high-pass filtered nuisance regressors were added to the FIR design as regressors of no interest.

#### 2.6.3 Cortical depth-specific HRF estimation and quantification

The voxel-wise HRFs estimated with the FIR were combined with the probability mask within the V1 ROI mask to obtain the HRF for each cortical depth per gas challenge. The cortical depth-specific HRF was defined as the weighted average of its depth probability and voxel-wise HRF. The benefit of using an FIR is the flexible estimation of a HRF as opposed to a basis function fit on the data, although it makes it more complicated to obtain quantitative descriptive parameters from the HRF. For HRF quantification and to mitigate noise from the data-driven FIR approach, a superposition of five inverse logit functions was fit to the laminae-derived HRF before quantification. Five inverse logit functions were chosen, because five can accurately fit an initial dip, transient sustained response peak, and undershoot, which is problematic when, for example, using three logit functions (Lindquist & Wager, 2007;Shan et al., 2014). Moreover, we did not opt for the use of the more frequently used double gamma function, since those may not provide accurate HRF fits for vasoactive stimulation. From the fitted HRFs we subsequently extracted the HRF parameters amplitude, time-to-peak (TTP), onset time, and full-width half-max (FWHM). TTP was defined as the time between stimulus presentation and peak amplitude. In the event of lack of detectable HRF (visually rated), the TTP was set to NaN. Onset time was defined as the intercept of the fitted line between 20% and 80% of the HRF amplitude and baseline (Tian et al., 2010). The quantification was performed on each subject’s laminae-specific HRFs.

#### 2.6.4 HRF CBVvO _2_ normalization

The amplitude of the BOLD signal is strongly dependent on venous CBV as evidenced by the literature on the M-value, reflecting the theoretical maximum BOLD signal change (Bandettini & Wong, 1995;Schellekens et al., 2023). Previous studies introduced a hypercapnic normalization of the BOLD signal to resolve the blood volume and CVR dependence (Bandettini & Wong, 1997;Cohen et al., 2004;Guidi et al., 2020;Liu et al., 2013). However, this relies on the iso-metabolic assumption that both CBV and CBF are elevated without altering the metabolic conditions during hypercapnia, which may not be true (Deckers et al., 2022;Driver et al., 2016,^2017^;Zappe et al., 2008). Therefore, an alternative normalization method based on hyperoxia was used in this study (Bulte et al., 2007;Chiarelli et al., 2007;Xu et al., 2012). Hyperoxia is a nonphysiological condition and other research has shown that similar hyperoxia levels as used in this study do not alter CBF, CaO_2_, substrate delivery, or cerebral metabolism (Ainslie et al., 2014). Using this assumption, that hyperoxia does not influence the metabolism and vasculature, the BOLD signal increase due to hyperoxia scales with the venous volume and the oxygenation. By using the ΔPetO_2_in the GLM to estimate$CBVv_{O_{2}}$, the effect of the oxygenation is minimized reflecting mostly the venous blood volume. Another reason to normalize with the baseline$CBVv_{O_{2}}$component only instead of additionally the dynamic CBV and CBF changes using CVR is that venous dilation only happens for prolonged stimuli resulting in overcompensation with the dynamic component in case of CVR normalization.

The amplitudes of the estimated HRFs were divided by the amplitude of the$CBVv_{O_{2}}$(%*BOLD/*∆*mmHg O_2_)*to account for the CBV dependence in the BOLD signal. It is expected that this normalization reduces the macrovascular influence on the BOLD signal and thus emphasizes the neuronal component of the HRF.

#### 2.6.5 Statistical analysis

Friedman tests, and Mack-Skillings tests in case of missing values for TTP, onset time and FWHM (Chatfield & Mander, 2009), were performed to compare how the sequence (GE and SE) and gas challenge influence the HRF as a function of cortical depth. In the event of missing values, the median (with 95% CI) is reported instead of the mean (± SD). This was followed by post hoc statistical tests to specifically investigate differences (a) within one lamina between sequences or (b) between two neighboring laminae for the same sequence. Holm-Bonferroni multiple comparison corrections were applied for the laminae comparisons. Additionally, a participant-wise linear fit was performed across cortical depth for all outcome variables separately and tested against the hypothesis of the slope being 0.

## 3 Results

### 3.1 CVR across cortical depth

**Fig. 4.**
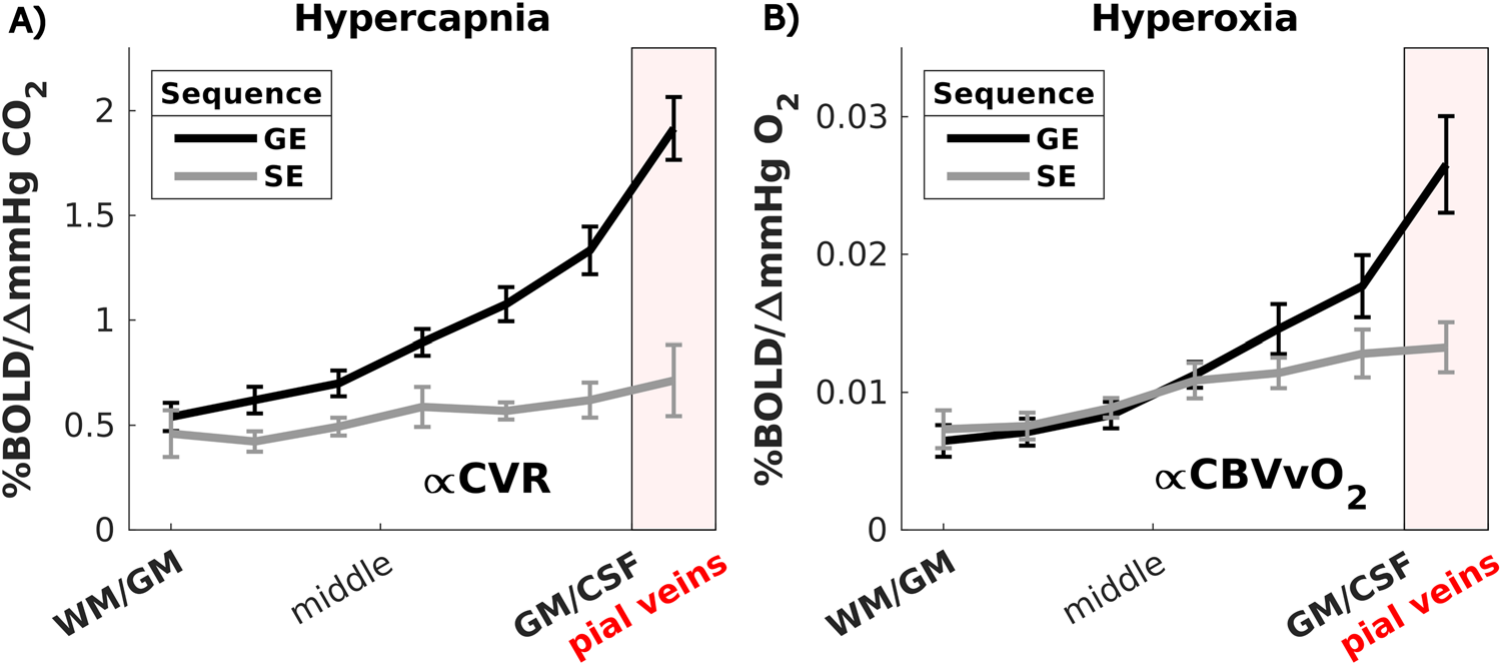
(A) CVR and (B)$CBVv_{O_{2}}$across cortical depth for the two scan sequences (GE and SE). The error bars refer to the standard error of the mean (SEM) across participants.

Mean increase in PetCO_2_across participants for the two hypercapnia (targets: +5 and +10 mmHg CO_2_) challenges was +4.7 ± 1.1 mmHg CO_2_and +8.6 ± 0.9 mmHg CO_2_, and PetO_2_increase during hyperoxia was +331 ± 33 mmHg O_2_(target: +350 mmHg O_2_). Achieved levels of ∆PetCO_2_and ∆PetO_2_are tabulated per participant in SupplementaryTable S1.Figure 4Ashows the CVR (in %/∆mmHg CO_2_) across cortical depth as measured with GE and SE. For GE (mean = 1.01 ± 0.2%/∆mmHg, t = 17.40, p < 0.001) and SE (mean = 0.55 ± 0.2%/∆mmHg, t = 7.63, p < 0.001), a significant CVR was found. When looking at all data over the laminae, CVR was higher as measured with GE than with SE (X^2^= 36, p < 0.001). CVR was different across cortical depth for GE (X^2^= 50, p < 0.001) and SE (X^2^= 15, p = 0.02). Post hoc tests revealed that GE CVR was larger in all laminae except the deepest laminae at the GM/WM border (post hoc z = 1.2, p = 0.110), where it was similar to SE. For GE, the CVR appears to be supralinearly increasing from deepest laminae at the GM/WM border (mean = 0.53 ± 0.2%/∆mmHg) toward the superficial laminae at the GM/CSF border and pial veins (mean = 1.92 ± 0.45%/∆mmHg) (0.21%/mmHg/laminae, CI = [0.15 to 0.28], F = 40.3, p = 0.0014). Conversely, for SE, it only increased from (mean = 0.46 ± 0.3%/∆mmHg) to (mean = 0.71 ± 0.5%/∆mmHg) around the large pial veins (0.04%/mmHg/laminae, CI = [0.008 to 0.08], F = 39.5, p = 0.0015). The reported slope of this increase in CVR across cortical depth was much larger for GE as compared with SE (post hoc z = 3.53, p = 0.004).

### 3.2 ***CBVvO _2_*** across cortical depth

The$CBVv_{O_{2}}$defined as the %∆*BOLD/mmHg O_2_*to the hyperoxic stimulus was used as proxy for baseline venous CBV and is shown inFigure 4B. For GE (mean = 0.013 %/∆mmHg, p < 0.001) and SE (mean = 0.010%/∆mmHg, p < 0.001), a significant increase in$CBVv_{O_{2}}^{GE}$and$CBVv_{O_{2}}^{SE},$respectively, was found due to hyperoxia as compared with baseline. When averaged over all laminae, no difference in$CBVv_{O_{2}}^{GE}$and$CBVv_{O_{2}}^{SE}$was found (X^2^= 2, p = 0.15), but cortical depth did explain substantial variance (X^2^= 54, p < 0.001). Examining laminae separately using post hoc analysis and multiple comparison correction, a difference in$CBVv_{O_{2}}^{GE}$and$CBVv_{O_{2}}^{SE}$reached significance only within the pial vein mask (post hoc z = 2.6, p = 0.04). The$CBVv_{O_{2}}$increased from deeper laminae toward the pial surface for GE (from 0.0065 +/- 0.0035% to 0.027 +/- 0.011%) (0.0031%/mmHg/laminae, CI = [0.0019 to 0.0043], F = 38.6, p < 0.0016) and SE (from 0.0073 +/- 0.0014% to 0.013 +/- 0.005%) (0.0011%/mmHg/laminae, CI = [0.0001 to 0.0021], F = 165.9, p < 0.001). This increase was larger for GE as compared with SE (post hoc z = 2.38, p = 0.0086) as can be seen by the steeper slope for GE inFigure 4B.

### 3.3 Visual task: HRF shape across cortical depth and gas challenges

**Fig. 5.**
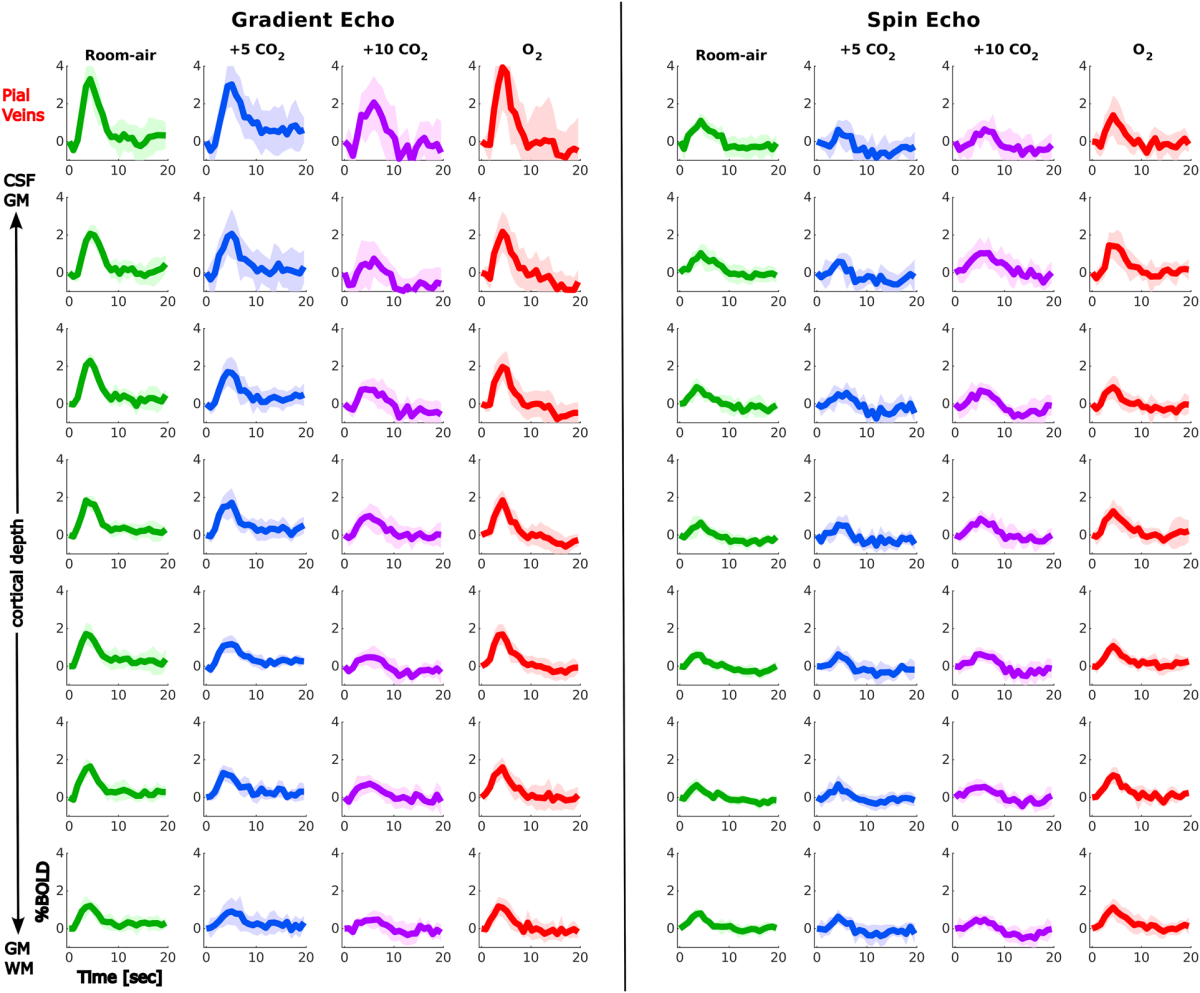
Mean non-normalized HRF to the visual task across participants, in % BOLD signal change, displayed across cortical depth (vertical) for the three gas challenges separately compared between sequences: GE (left) and SE (right), colors indicate the different gas blocks during which the HRF was estimated. Shading refers to the 95% confidence interval across participants. The GE HRF amplitude increases toward the pial surface and decreases with increasing hypercapnia level where hyperoxia seems to have no effect. Hypercapnia does not seem to have an effect on the SE HRF amplitude. GE HRF amplitude is overall much larger than SE.

Figure 5shows the non-normalized mean HRFs to the visual task across participants for GE (left panel) and SE (right panel) during the different gas challenges (colors) and across cortical depth (vertical). The HRF at room air shows the expected shape peaking around 3-5 s after stimulus onset. The HRF shape changes with increasing hypercapnia levels seen by lower amplitudes and wider peaks. Moreover, the HRF shapes become noisier during hypercapnia and to a lesser extent hyperoxia, reflected by larger confidence intervals as can be seen inFigure 5. During the*O*_2_challenge, the HRF shape returns to the shape during room air.

#### 3.3.1 HRF amplitude

Quantified HRF amplitudes are shown inFigure 6A. The HRF amplitudes (%∆BOLD) during room air are twice as large for GE (mean = 2.17 ± 0.9%∆BOLD) compared with SE (mean = 0.90 ± 0.5%∆BOLD) (X^2^= 55.3, p < 0.001). This holds also for the hypercapnia gas challenges (+5 mmHg) (X^2^= 38.16, p < 0.001) and (+10 mmHG) (X^2^= 5, p = 0.25), and hyperoxia (X^2^= 26.8, p < 0.001), see SupplementaryTable S2.

**Fig. 6.**
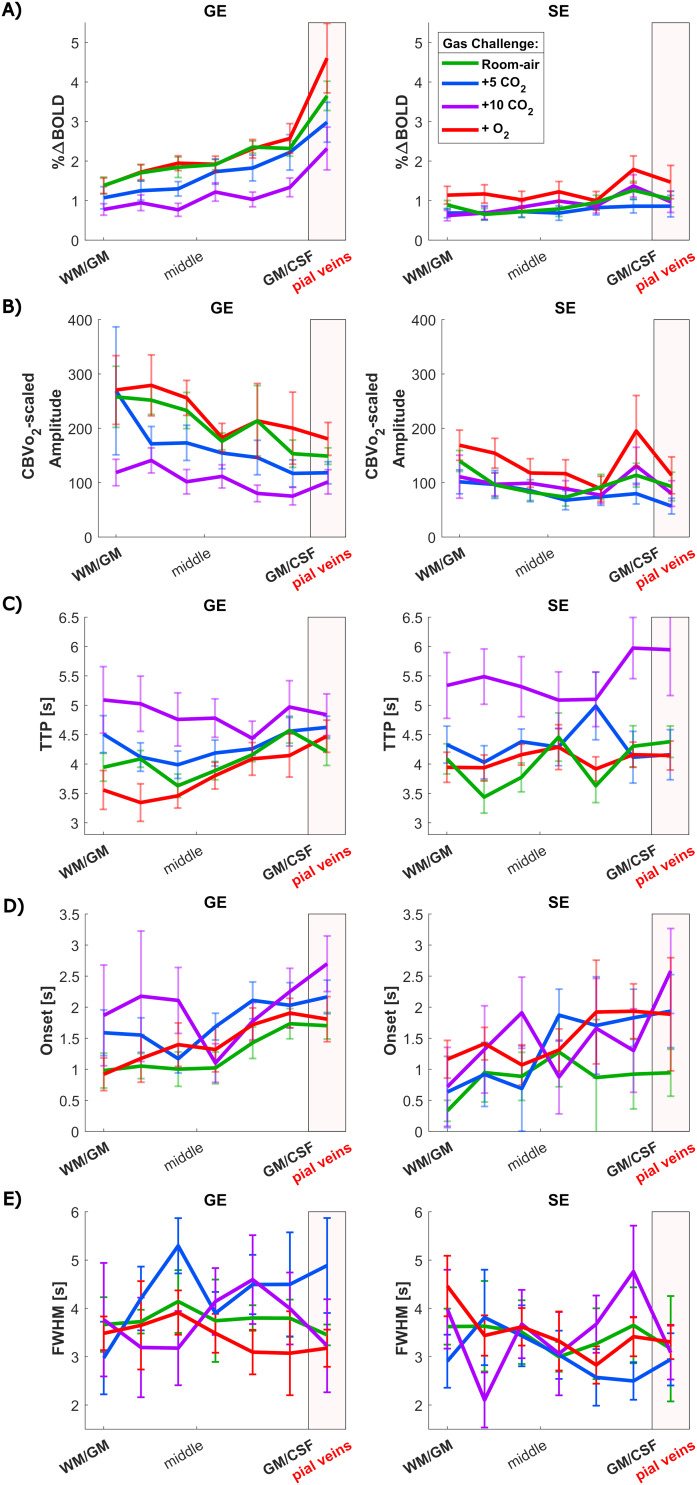
HRF quantification results: (A) amplitudes, in % BOLD signal change, (B) amplitudes normalized for CBV dependence by the$CBVv_{O_{2}}$, (C) TTP, (D) onset time, and (E) FWHM. All displayed across cortical depth for the four gas challenges separately compared between sequences: GE-BOLD (left) and SE-BOLD (right), colors indicated different gas challenges. The error bars refer to the standard error of the mean (SEM) across participants (n = 9).

The gas challenge explained most of the variance observed in the amplitudes (X^2^= 39, p < 0.001). During hyperoxia, the HRF amplitudes for GE (mean = 2.35 ± 1.5%∆BOLD) and SE (mean = 1.26 ± 0.9%∆BOLD) are comparable with the room-air condition (post hoc z = -1.50, p = 0.93). In contrast, during hypercapnia, the GE HRF amplitudes are reduced (X^2^= 38, p < 0.001) to 1.77% for +5 mmHg (post hoc z = 2.7, p = 0.0033) and 1.20%∆BOLD for +10 mmHg (post hoc z = 6.1, p < 0.001). For SE, no significant effect of hypercapnia on the HRF amplitude was observed (X^2^= 3.4, p = 0.19).

The from literature well-known GE HRF amplitude increase toward the pial surface can also be observed inFigure 6A. The GE amplitudes steadily increased from the deeper GM toward the pial veins during room air (0.31%/laminae, CI = [0.15 to 0.45], F = 20.0, p = 0.020), +5 mmHg CO_2_(0.29%/laminae, CI = [0.14 to 0.44], F = 46.7, p = 0.0041), +10 mmHg CO_2_(0.20%/laminae, CI = [0.05 to 0.36], F = 9.9, p = 0.030), and hyperoxia (0.41%/laminae, CI = [0.17 to 0.66], F = 13.0, p = 0.030). For SE, no increasing amplitude trend toward the pial surface was observed (0.070%/laminae, CI = [-0.0008 to 0.14], F = 4.86, p = 0.16), reflecting the more homogeneous distributed microvasculature across cortical depth compared with the macrovasculature. The difference in amplitude between GE and SE increased from the deeper GM toward the pial surface (post hoc z = 2.73, p = 0.0248), and can visually be observed by comparingFigure 6Aleft and right.

#### 3.3.2 Normalized/scaled HRF amplitude

To account for the CBV dependence in the HRF amplitudes, they were normalized by the$CBVv_{O_{2}}$(Fig. 4B), as visualized inFigure 6B. The cortical depth profile changed, and even reversed, due to this normalization. The normalized GE HRF amplitude decreased from the deeper GM toward the pial surface during room air (-0.060[a.u.]/laminae, CI = [-0.087 to -0.032], F = 34.7, p = 0.008). While for SE it might be observed visually, no statistically significant trend across cortical depth was observed (-0.021[a.u.]/laminae, CI = [-0.070 to 0.027], F = 1.20, p = 0.55). However, the difference between GE and SE trends across cortical depth is no longer significantly different (post hoc z = -1.32, p = 0.74) after normalization, meaning that the cortical profile of GE starts to become similar as SE. After normalization, we do not observe a difference across cortical depth with GE during the highest hypercapnia level (-0.040[a.u.]/laminae, CI = [-0.0840 to 0.0052], F = 5.34, p = 0.074), giving an equal cortical profile as SE as can be seen inFigure 6Bfor all gas challenges.

#### 3.3.3 HRF temporal features

The HRFs were characterized by the quantitative descriptors TTP, onset time, and FWHM, which are visualized inFigure 6C-E. A similar range of TTP was found for GE (median = 4.24 s, CI = [3.63 to 4.86]) and SE (median = 4.29 s, CI = [3.49 to 5.08) combining all laminae and all gas challenges, with no statistical difference between the acquisition methods (t = 0.28, p = 0.59). A Mack-Skilling’s test was performed to test whether the gas challenges predict variance in the TTP for GE and SE separately. A clear difference in TTP was found between gas challenges for both GE (t = 27.3, p < 0.001) and SE (t = 34.4, p < 0.001). TTP was significantly higher for GE for both hypercapnia levels, respectively (median = 4.28 s, CI = [3.81 to 4.75]) (post hoc z = -2.5, p = 0.011), and (median = 4.84 s, CI = [4.04 to 5.64]) (post hoc z = -3.9, p < 0.001) compared with room-air breathing (median = 4.09 s, CI = [3.69 to 4.49]). For SE, we only observed an increase in TTP for the highest hypercapnia level (median = 5.4 s, CI = [4.40 to 6.43]) compared with the room-air condition (median = 3.96 s, CI = [3.36 to 4.55]) (post hoc z = -4.2, p < 0.001). Hyperoxia was not observed to influence TTP compared with room air (post hoc z = -0.47, p = 0.32) independent of acquisition sequences. A linear increase in TTP from the deep GM toward the pial veins can be seen for GE (0.075s/laminae, CI = [0.003 to 0.15], t = 2.31, p = 0.025) and SE (0.096s/laminae, CI = [0.022 to 0.17], t = 3.0, p = 0.009) during room-air breathing with no difference between the trends for the two sequences (post hoc z = -0.17, p = 0.86). During the hypercapnia conditions, this trend toward the pial surface was not observed. In other words, HRFs were delayed with increasing CO_2_levels, and this delay was similar across cortical depth for both GE and SE.

No difference in onset time (Fig. 6D) was found between GE (median = 1.61 s, CI = [0.84 to 2.37]) and SE (median = 1.30, CI = [0.15 to 2.45]) combined over all laminae and gas challenges (t = 3.13, p = 0.077). However, the onset time changed significantly between the three gas challenges and room air (t = 15.2, p = 0.0016). Taking the latter into account, we found that SE (median = 0.90 s, CI = [-0.17 to 1.98]) had significantly faster onset time during room air compared with GE (median = 1.30 s, CI = [0.79 to 1.82]) (t = 8.11, p = 0.018). For hypercapnia and hyperoxia, no difference in onset time between GE and SE was found. The onset time across cortical depth is visually increasing from the WM/GM-border toward the pial surface for GE (0.14s/laminae, CI = [0.048 to 0.24], F = 22.49, p = 0.021) and SE (0.12s/laminae, CI = [0.017 to 0.23], F = 1.29, p = 0.78) during room-air breathing, but did not reach significance for SE. We found the same trends across cortical depth for the hyperoxia gas challenge (post hoc z = 0.088, p = 0.53), GE (0.16s/laminae, CI = [0.051 to 0.27], F = 20.7, p = 0.030), and SE (0.22s/laminae, CI = [-0.054 to 0.49], F = 5.63, p = 0.35). For the hypercapnia challenges, these cortical trends do not reach significance but can be observed visually inFigure 6D.

FWHM was found to be higher for GE (median = 3.66 s, CI = [2.3 to 5.0]) compared with SE (median = 3.3 s, CI = [2.0 to 4.6]) (t = 15.41, p < 0.001) combined over all laminae and gas challenges, meaning longer HRFs were found with GE (Fig. 6E). No difference in FHWM was found between gas challenges for all cortical depths (t = 1.12, p = 0.77). Statistical tests across cortical depth for FWHM were not performed because it was difficult due to noise in single HRF data, to accurately estimate the FWHM at deeper layers during hypercapnia for all subjects.

## 4 Discussion

### 4.1 General discussion

In this study, we investigated the contribution of vascular properties to BOLD signals across cortical depth that arise from neuronal activity or vascular-related processes. Neuronal activity-induced HRFs were estimated using short event-related visual stimuli, while vascular-related hemodynamic changes were elicited by inhalation of different levels of CO_2_and O_2_. BOLD responses were obtained across cortical depth using high spatiotemporal resolution 7T MRI, and sensitizing the signal toward microvessels or all vessels was achieved by SE and GE fMRI, respectively.

As expected, we observed that GE-BOLD contains contributions from all venous vessel sizes evidenced by the well-known HRF amplitude increase toward the pial surface. This increase was not present in SE-BOLD (Fig. 6A). This behavior can be explained by the increase in vessel diameter toward the pial surface (Hirsch et al., 2012), which was also supported by the observed increase in$CBVv_{O_{2}}$toward the pial surface. Thus, our current laminar data align with predictions from recent hemodynamic synthetic vascular simulations (Havlicek & Uludag, 2020;Markuerkiaga et al., 2016), which demonstrate larger BOLD signal changes toward the pial surface compared with deeper layers for GE acquisitions, as well as differences in temporal HRF properties, accounting for the contribution of angioarchitecture. The simulation inUludağ and Blinder (2018)showed a higher SE signal compared with GE but not accounting for AVs that would yield an additional linear increasing signal change from the deep GM toward the pial surface and thereby line with the in vivo results presented here.

Scaling the HRF amplitudes by$CBVv_{O_{2}}^{GE}$and$CBVv_{O_{2}}^{SE}$to account for relative CBV differences across cortical depth resulted in a similar cortical HRF amplitude profile for GE and SE. This might mean that the normalized HRF amplitudes may better reflect the microvascular BOLD signal or that the signal changes of all vascular compartments are relatively similar when accounting for$CBVv_{O_{2}}$differences. Additionally, we found that CO_2_-induced dilation has limited effect on BOLD signals assumed to be mainly originating from microvasculature, as we observed (1) a limited effect of increasing levels of hypercapnia on the SE HRF amplitude for all cortical depths and (2) a similarly limited effect on the GE HRF amplitude in the deeper GM. This suggests that the influence of vasoactive effects on visually induced BOLD signals from the microvasculature is possibly negligible and that such signals reflect neuronal processes. In contrast, the hyperoxia task did not affect the visually evoked HRFs that were similar to those while subjects breathed room air.

### 4.2 Vascular effects

#### 4.2.1 CBVvO _2_ increase toward the pial surface

$CBVv_{O_{2}}^{GE}$and$CBVv_{O_{2}}^{SE}$, estimated using the hyperoxia task, were defined as a measure of relative resting CBV. Resting CBV relates only to the vascular density and diameters and can introduce a corresponding bias in the amplitude (and shape) of the BOLD signal measurements. Here we estimated the$CBVv_{O_{2}}$by means of a hyperoxia challenge and examined the contribution of this bias for GE and SE across cortical depth. The basic underlying assumption is that$CBVv_{O_{2}}^{GE}$for GE reflects the blood volume of veins of all sizes, whereas$CBVv_{O_{2}}^{SE}$for SE reflects mainly microvessels. We found that both$CBVv_{O_{2}}^{GE}$and$CBVv_{O_{2}}^{SE}$increased toward the pial surface (Fig. 4). This was expected for GE but not for SE as the capillary network is assumed to be more homogeneously distributed across cortical depth (Siero et al., 2013). From this, it can be concluded that SE also exhibits some sensitivity toward larger veins in the superficial laminae possibly due to R2’ effects in the SE-EPI readout (Birn et al., 2008;Goense & Logothetis, 2006;Van Horen et al., 2023). Additional increased R2’ weighting during hyperoxia in the SE could be related to two signal component effects: (1) the extravascular component due to a shift in SE sensitivity toward slightly larger vessels because of smaller susceptibility differences ascribed to higher venous oxygen saturation (Boxerman et al., 1995) and (2) The increased intravascular component originating from both arteries and veins in a hyperoxia state remains significant in spin echo acquisitions, particularly at high oxygen saturation levels. Difference between$CBVv_{O_{2}}^{GE}$and$CBVv_{O_{2}}^{SE}$did not reach significance in the defined laminae except the pial veins, although visually, a difference can be seen inFigure 4Bin the superficial laminae around the pial surface. The similarity between$CBVv_{O_{2}}^{GE}$and$CBVv_{O_{2}}^{SE}$indicates that GE and SE are mainly sensitive to the same vasculature in the deep and middle GM laminae, mainly microvasculature, that is predominately located at this cortical depth (Hirano et al., 2011). This finding also implies that CBV contributions to BOLD in response to a visual task are predominately from microvessels in the deeper laminae. On the contrary, we found that differences in$CBVv_{O_{2}}$between GE and SE increased toward the pial surface. This implies that toward the pial surface, the relative$CBVv_{O_{2}}^{GE}$contribution and overall volume of the macrovasculature increase and exceed that of the microvasculature. Accounting for these$CBVv_{O_{2}}$biases, for example, with normalization methods, as inSection 3.3.3, or modeling (Báez-Yáñez et al., 2020,^2023^;Havlicek et al., 2015;Markuerkiaga et al., 2016), can help to obtain cortical depth profiles more specific to the microvasculature, and thereby to the underling neuronal activity. One might expect a peak in$CBVv_{O_{2}}^{SE}$around the middle laminae as previously have been shown in high resolution BOLD and VASO data (Fracasso et al., 2018;Koopmans et al., 2010), because of high vascular density in layer IV, which is not observed here. We think the reason for this might be the acquired resolution (1.5 mm) that is not sufficient to find that “bump” around layer IV due to partial volume effects and taking into account the relatively small thickness of the visual cortex (Duvernoy et al., 1983). Additionally, the laminae based on cortical depth used in this work correspond not directly with true layers that can be distinguished using histology.

#### 4.2.2 Hypercapnic gas challenge mainly affects macrovasculature

CVR was estimated using the vasoactive hypercapnia challenge. Voxels or regions with high CVR are likely to also show a large vasoactive component in the neurovascular evoked hemodynamic response (Schellekens et al., 2023). The CVR was found to be larger for GE compared with SE. This difference increased toward the pial surface (Fig. 4), implying that hypercapnia mainly affects the larger vessels and to a lesser extent the microvasculature, also supported by the observed coinciding$CBVv_{O_{2}}^{GE}$increase (Fig. 4). This may suggest that vessel reactivity as measured by GE-BOLD is proportional to vessel size and density. Another factor that may contribute to the difference in CVR between GE and SE could be the cellular composition of the vessel wall of capillaries versus larger vessels. Capillaries mostly consist of endothelial cells with tight junctions, and some might contain contractile pericytes. Arteries and to a lesser extent veins are additionally covered by a thin smooth muscle sheath, which is known to regulate vessel diameter (Hall et al., 2014;Schmid et al., 2019). If capillaries react differently to hypercapnic vasodilation than postcapillary and larger vessels, this could result in different CVR between SE and GE at deeper cortical depths while$CBVv_{O_{2}}$is similar for SE and GE. Dilation in the microvasculature seems limited as the SE CVR is low and as it shows a much weaker trend across cortical depth than GE. This suggests that the HRF as measured with SE should contain negligible purely vasoactive signal changes, supported by the absence of smooth muscle cells around capillaries.

### 4.3 Neurovascular effects

#### 4.3.1 Effects of hypercapnia and hyperoxia on HRF amplitude

No difference in HRF amplitude between room air and hyperoxia was found for both sequences, in agreement with reports that this hyperoxia level is not influencing the hemodynamics (Ainslie et al., 2014). The similarity in HRF amplitudes between room air and hyperoxia is also in line with electrophysiology findings (Groen et al., 2022), indicating that there is no habituation to the visual stimuli during the period of a functional run, considering that the room air and hyperoxia occurred in the beginning and end of the run, respectively. We observed that the amplitude of the SE HRF in response to the visual task was not much affected by hypercapnia. Therefore, we argue that the microvascular HRFs estimated with SE are influenced less by sustained vasodilation (as induced by CO_2_) and rather respond to neuronal activation. For GE-BOLD, we found a decrease in HRF amplitude with increasing CO_2_levels. These observations are in line with observations by Gauthier et al., during carbogen breathing experiments (hypercapnia and hyperoxia combined) at 3T (Gauthier et al., 2011). Several aspects can potentially explain this decrease in GE HRF amplitude: (1) Dilatory capacity is already reduced due to hypercapnia resulting is only small additional visual stimuli-induced dilation. (2) Increased basal levels of CBV and CBF leading to saturation of a neuronally evoked BOLD signal reducing its amplitude (Brown et al., 2003;Siero, Hartkamp, et al., 2015). (3) Any temporal mismatch between the underlying CBV, CBF, and CMRO_2_responses also determines the BOLD response shape. All of which, individually, can be modulated by hypercapnia to a varying degree (Siero, Hendrikse, et al., 2015).

Because a decrease in HRF amplitude is only observed with GE and not SE, it can be ascribed to macrovascular changes. This same argument can be used to ascribe this change to the vasculature instead of decreased metabolic activity during hypercapnia, otherwise a decrease in SE HRF amplitude would be expected as well. However, it could be that the SE signal from the microvasculature is too low to pick up any variation due to hypercapnia or that the physiological noise is higher in these compartments, overwhelming any hypercapnia-induced variance. Moreover, it should be noted that the resting state network activity might be reduced during hypercapnia (Driver et al., 2016,^2017^). These results suggest that vessel reactivity of larger intracortical veins (and pial vessels) is reduced when vessels are already predilated due to the hypercapnic condition. Additionally, the vasodilation during hypercapnia leads to an increase in CBV and CBF, which results in a BOLD signal increase by itself as seen inFigure 4(Bandettini & Wong, 1995).

#### 4.3.2 CBV contribution to HRF amplitude across cortical depth

The well-known increase in the GE HRF amplitude toward the pial surface was also observed in our data. This is in agreement with previous studies (Bause et al., 2020;Polimeni et al., 2010;Ress et al., 2007;Siero et al., 2013), but our study expanded by explicitly examining the HRF around pial veins. Similarly as in the CVR, the visually evoked increase in GE HRF amplitude coincided with the increase of$CBVv_{O_{2}}^{GE}$across cortical depth estimated from the hyperoxia challenge. In contrast to GE, the SE data showed no difference in HRF amplitude across cortical depth, plausibly caused by the more homogeneous distribution of the capillary bed (Duvernoy et al., 1981;Kim et al., 2013;Weber et al., 2008). Especially the finding that the pial veins do not have a higher SE HRF amplitude confirms that SE during room-air breathing is weighted toward the microvasculature and is, therefore, more specific to neuronal activity. We normalized the HRF amplitudes for their CBV dependence using$CBVv_{O_{2}}$. No difference in slope across cortical depth for the normalized HRF amplitude was found between GE and SE (Fig. 6B), meaning that their cortical depth profile became similar. This implies that the CBV contribution of the macrovasculature is relatively reduced after this normalization, and, therefore, it might reflect the microvasculature better. As it is believed that the microvascular response is closest to the neuronal response, we argue here that the normalized signal changes reflect the neuronal component of the visual task better by reducing the CBV dependence. This makes it preferable over the non-normalized GE HRF for investigation of brain functions. We observed a small decrease of the normalized HRF amplitude toward the pial surface, which is the inverse compared with the non-normalized amplitudes. This suggests that microvessels may generate relatively more signal than the larger veins, if CBV biases are accounted for (Schellekens et al., 2023). This effect is in agreement with previous research byGuidi et al. (2016), where they normalized the HRF amplitude by the CVR. Alternatively, this could also suggest that the neurovascular signals in the macrovasculature may be attenuated by the large effects that CBV differences across cortical depth have on BOLD. Another reason could be that the normalization using$CBVv_{O_{2}}$compensates more in the superficial layers and pial surface because venous dilation primarily happens for prolonged stimulations. However, the exact reason for the observed decrease in normalized HRF amplitude toward the pial surface remains an open question and needs to be further investigated. Similarly as in the$CBVv_{O_{2}}^{SE}$, we did not observe a peak in normalized GE HRF amplitudes in the middle of the cortex related to layer IV probably because of insufficient voxel size.

#### 4.3.3 Effect of gas challenges on HRF TTP and onset time across cortical depth

The amplitude of the HRF differed highly between GE- and SE-BOLD, but we found no significant difference in TTP between the two sequences. This is in contrast with results from previous research where a shorter TTP was found for SE (Siero et al., 2013). However, we did observe a faster onset time for SE. Additionally, we did find an increase in TTP from the deeper GM toward the pial surface for GE during room-air breathing, which was also observed bySiero et al. (2013). This can be explained by the earlier dilation of the arteries in the deeper layers (Tian et al., 2010). Another possible reason for the increase in TTP toward the pial surface is the drainage of deoxygenated blood through the macrovasculature to the pial surface, in line with the observation that the onset time was found to be faster for the microvasculature as measured by SE than for GE. However, the latter explanation seems to be less plausible since the increase in TTP across cortical depth is also observed for SE that primarily senses the microvasculature. The vascular manipulation by hypercapnia may also impact this result as discussed below.

Longer TTPs were found during hypercapnia compared with room-air breathing, meaning that a delayed or prolonged response was observed (Fig. 6C). This increase in TTP in combination with the aforementioned decrease in GE HRF amplitude due to*CO*_2_inhalation suggests that vascular reactivity of the macrovasculature is decreased during hypercapnia. A similar increase in TTP was found in the SE-BOLD scans. One possible explanation could be that vessels are not at their resting tone during hypercapnia, since they are predilated, and thus any further dilatory stimulus may be slowed considering the nonlinear BOLD response to CO_2_(Bhogal et al., 2014). We can hypothesize that the mechanical properties and basal CBF and CBV level of the vessels are altered, and this propagates into the hemodynamic response. Another argument that can be made is that CMRO_2_is depressed under hypercapnia. So, differences in TTP may also be a result of a changed metabolic state, or temporal mismatch between CBF and CBV responses in time (Siero, Hendrikse, et al., 2015), rather than relating to the potency of dilation. Lastly, a reason could be that the increase in TTP relates to an overall increase in the duration of the hemodynamic response, compensating for the reduced OEF with increased CBF and possible increased capillary transit time heterogeneity to allow for adequate oxygen delivery (Angleys et al., 2018;Jespersen & Ostergaard, 2012). However, we did not observe a sustained or longer HRF that would have been reflected in the FWHM, possibly because of the noisiness of the estimation of the FWHM.

The longer TTP and lower amplitude during hypercapnia imply that care must be taken in interpreting the BOLD signal as a measure of neuronal activity in subjects with altered baseline CBF or after intake of substances with vasodilatory properties. The HRFs estimated from the visual task during hypercapnia are noisier compared with room air, possibly also influencing the TTP estimations. A potential reason is the motion associated with the discomfort of the hypercapnic breathing task that presented itself in more pronounced nodding. Moreover, more visual stimuli were presented during baseline (17 stimuli) than during each hypercapnic level (≈10), giving less statistical power for the FIR analysis during hypercapnia.

### 4.4 Methodological considerations

In the performed 7T BOLD-fMRI experiments, both temporal and spatial resolution were pushed and a limited FOV was required to maintain a short TR. The small FOV complicates retrospective motion correction of the functional scans and registration with structural scans (Friston et al., 1996). In this study, motion correction was even more complicated because of the extra head motion introduced by relatively deeper breathing due to discomfort of hypercapnia. Additionally, the motion correction algorithm may misinterpret the BOLD effect, for example, from hypercapnia, as motion (Schulz et al., 2014).

A second consideration is that a lower spatial resolution was used for SE to gain SNR to match the GE SNR, which results in more partial volume effects. Ideally, the voxel size for the two sequences would be similar for a more optimal comparison across the cortical depth. However, smaller voxel sizes would also result in an increase in the EPI-readout length under the constraint of the used subsecond TR. A longer EPI train will lead to additional T2* contamination in the signal for SE and, therefore, more unwanted weighting toward the macrovasculature. Additionally, the laminar specificity might be compromised for SE compared with GE because of the lower resolution of 1.5 mm given an approximate cortical thickness of 2 mm in the visual cortex (Duvernoy et al., 1983). The use of probability masks in this work partly corrects for the partial volume effects within laminae.

InFigure 2A, a Pet-CO_2_time course of a representative subject is shown. A transient in Pet-CO_2_can be observed after the second hypercapnia period (400:500 s), possibly induced by panting. This may result in Pet-CO_2_-induced changes in the vasculature during the hyperoxia period resulting in additional measurement noise.

## 5 Conclusion

Quantification of the contribution of different vascular compartments to the BOLD signal is essential to interpret studies of neuronal activation on the laminar scale. The BOLD signal from the microvasculature, estimated from SE-BOLD, was less influenced by purely vascular processes as seen by low CVR and no HRF amplitude change during hypercapnia as examined in this study. These observations combined suggest that the visual stimulus-induced SE-BOLD signal originating from the microvasculature is less affected by vascular processes in comparison with GE-BOLD. We observed an increase in GE HRF amplitude toward the pial surface, which coincided with an increase in CVR and$CBVv_{O_{2}}^{GE}$. Additionally, we found an increase in TTP from the deep GM toward the pial surface during room-air breathing for the microvasculature (SE) as well as for all venous compartments (GE). For GE, we also observed an increase in onset time from the deeper laminae toward the pial surface, showing faster onset in the deeper laminae. Therefore, our data suggest that the$CBVv_{O_{2}}^{GE}$distribution across cortical depth, which was estimated with the hyperoxia task, in addition to the CVR, is the main contributor to the observed GE HRF amplitude increase. Lastly, we showed that it is possible to partially correct for this$CBVv_{O_{2}}$by using hyperoxia normalization resulting in similar cortical depth profiles for GE- and SE-BOLD.

## Supplementary Material

Supplementary Material

## Data Availability

All data will be accessible through Flywheel. Code will be shared upon reasonable request.
