# Supplementary material for: The contribution of the vascular architecture and cerebrovascular reactivity to the BOLD signal formation across cortical depth
Source: Imaging Neurosci (Camb). 2024 Jun 28;2:imag-2-00203. doi: 10.1162/imag_a_00203 (PMC11472217; doi:10.1162/imag_a_00203)
Supplement: Supplementary Material [file imag_a_00203-supp.pdf]

Supplementary

Figure S1. Example showing the two pial veins segmentation tools to be complementary. Note that the union shows better connected veins. Green = Nighres, Red = BrainCharter.

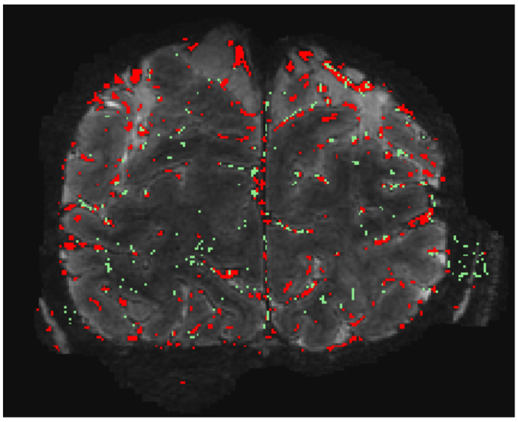

Table S1. **Average obtained PetCO$_2$ and PetO$_2$ values.**

	+5 mmHg CO_2		+10 mmHg CO_2		+350 mmHg O_2	
	**GE**	**SE**	**GE**	**SE**	**GE**	**SE**
**sub02**	2.7	2.2	7.4	7.7	372.9	369.8
**sub03**	4.4	4.2	7.4	7.9	354.2	338.8
**sub04**	5.1	5.2	9.0	8.4	287.8	305.7
**sub06**	4.5	4.9	8.7	8.4	366.2	350.9
**sub07**	5.8	5.1	8.9	8.1	364.9	350.9
**sub08**	6.3	6.2	10.2	10.3	350.6	343.9
**sub09**	5.8	5.7	10.0	9.5	373.4	372.3
**sub10**	5.0	5.3	8.5	8.7	298.5	317.2
**sub11**	4.3	4.3	7.8	8.0	282.6	278.5

Table S2. **HRF amplitudes average across cortical depth**

HRF amplitude [%ΔBOLD] (mean+sd)	Gradient Echo (GE)	Spin Echo (GE)
**Room-air**	2.1656 ± 0.9495	0.9001 ± 0.5326
**Hypercapnia +5 mmHg CO_2**	1.7683 ± 1.1649	0.7598 ± 0.5314
**Hypercapnia +10 mmHg CO_2**	1.1978 ± 0.9278	0.9040 ± 0.6236
**Hypercapnia +350 mmHg CO_2**	2.3498 ± 1.5135	1.2569 ± 0.8549
